# Cobalt Impregnation on Titania Photocatalysts Enhances Vis Phenol Photodegradation

**DOI:** 10.3390/ma16114134

**Published:** 2023-06-01

**Authors:** Soukayna Belekbir, Mohammed El Azzouzi, Laura Rodríguez-Lorenzo, Adnane El Hamidi, Juan Arturo Santaballa, Moisés Canle

**Affiliations:** 1 Laboratory of Nanomaterials, Nanotechnologies and Environment, Center of Materials Science, Faculty of Sciences, Mohammed V University in Rabat, Rabat BP 1014, Morocco; 2 INL—International Iberian Nanotechnology Laboratory, Water Quality Group, Av. Mestre José Veiga, 4715-330 Braga, Portugal; 3 React! Group, Department of Chemistry, Faculty of Sciences & CICA, University of A Coruña, E-15071 A Coruña, Spain

**Keywords:** photocatalysis, phenol, cobalt, oxygen vacancies, UV-Vis radiation (366 nm), UV (254 nm), TiO_2_-P25, wet impregnation, SERS

## Abstract

One of the main challenges of photocatalysis is to find a stable and effective photocatalyst, that is active and effective under sunlight. Here, we discuss the photocatalytic degradation of phenol as a model pollutant in aqueous solution using NUV-Vis (>366 nm) and UV (254 nm) in the presence of TiO_2_-P25 impregnated with different concentrations of Co (0.1%, 0.3%, 0.5%, and 1%). The modification of the surface of the photocatalyst was performed by wet impregnation, and the obtained solids were characterized using X-ray diffraction, XPS, SEM, EDS, TEM, N_2_ physisorption, Raman and UV-Vis DRS, which revealed the structural and morphological stability of the modified material. BET isotherms are type IV, with slit-shaped pores formed by nonrigid aggregate particles and no pore networks and a small H3 loop near the maximum relative pressure. The doped samples show increased crystallite sizes and a lower band gap, extending visible light harvesting. All prepared catalysts showed band gaps in the interval 2.3–2.5 eV. The photocatalytic degradation of aqueous phenol over TiO_2_-P25 and Co(X%)/TiO_2_ was monitored using UV-Vis spectrophotometry: Co(0.1%)/TiO_2_ being the most effective with NUV-Vis irradiation. TOC analysis showed ca. 96% TOC removal with NUV-Vis radiation, while only 23% removal under UV radiation.

## 1. Introduction

Many activities, such as agriculture, medicine, or industry, release pollutants into the environment, creating one of the most serious problems we currently face: environmental pollution. Water pollution, in particular, poses important risks to human health and the environment. Therefore, the control of pollutants released into aqueous media has led to increasingly restrictive environmental regulations. Phenol is considered a major pollutant because of its toxicity, bioaccumulation, low biodegradability, and carcinogenicity [1]. It causes an unpleasant taste and odor in drinking water [2], and it is harmful to living organisms at low concentrations, toxic and mutagenic, being absorbed through the skin at high concentrations [3]. The main sources of anthropogenic phenols in the aquatic environment are wastewaters from manufactures of resins, chemicals, dyes, polymers, pesticides, coal conversion, and petrochemical industries [4].

Considerable efforts have been devoted to developing approaches aiming to a complete removal of organic pollutants. However, the application of conventional methods, such as membrane filtration, adsorption on activated carbon, ion exchange on synthetic resins, purification by chemical coagulation systems, etc., are not cost effective and may generate wastes that require further treatment stages and additional costs [5,6]. Conversely, photocatalysis requires low cost and abundant chemicals, at little or no energy expense, and can operate autonomously [7], requiring only light (Visible and/or UV) and a semiconductor catalyst to achieve or accelerate chemical reactions [8,9,10,11,12,13]. Though a number of photocatalysts have been studied (Al_2_O_3_, ZnO, Fe_2_O_3_, and TiO_2_) [14], the most widely used one is TiO_2_, of which the photoactive forms are anatase and rutile. One of the most effective forms, and by far the most widely used, is a mixture of anatase/rutile at a ratio of ca. 80%/20%, with a small percentage of amorphous form, commercialized as Evonik-P25 (referred to here as P25) [15]. The improved photocatalytic activity of P25 is attributed to its junction, which reduces the e^−^/h^+^ recombination [16]. TiO_2_-P25 is known for its lack of toxicity when presented as aggregates or macroparticles, chemical stability in aqueous solution, and low cost [17]. Despite all these advantages, its use remains limited because of its high band-gap energy, 3.2 eV, thus limiting its photocatalytic activity to the UV region, which represents only 4 to 5% of the solar spectrum.

Numerous strategies have been used to increase the photocatalytic efficiency of TiO_2_-P25 under Vis irradiation, including doping/loading with metal ions or nonmetals [18,19]. Doping allows a reduction in the e^−^/h^+^ recombination and decreases the band gap, shifting the photocatalyst light absorption towards the Vis region, which facilitates the use of sunlight as the irradiation source [20]. Recently, a number of papers have been devoted to the photodegradation of phenolic compounds [21]; many of them related to the application of TiO_2_ as a photocatalyst [22,23]. Co(II)-doped TiO_2_ was found to have high photocatalytic activity for the degradation of acetaldehyde [24], 2-chorophenol [25], azo dyes [26], and even exhibited antimicrobial features [27].

Here, we present an unprecedented extensive study of phenol photodegradation under UV and near-visible light irradiation, using TiO_2_-P25 (Degussa) loaded with Co ions at different concentrations (Co(X%)/TiO_2_, where X = 0.1%, 0.3%, 0.5%, and 1%). The samples were prepared by the wet impregnation method. Extensive characterization of the samples was carried out using X-ray diffraction (XRD), SEM, EDS, TEM, X-ray photoelectron spectroscopy (XPS), Raman and UV-Vis diffuse reflectance spectroscopy (UV-Vis DRS), and N_2_ physisorption. Its photocatalytic activity was evaluated by monitoring phenol transformation, taken as a model organic pollutant, and total organic carbon (TOC) evolution. The photoreaction was conducted in an aqueous solution under near-UV light at wavelengths of 366 nm and longer and also under UV light (254 nm).

## 2. Experimental Procedure

### 2.1. Materials

TiO_2_-P25 was from Evonik (ca. 70:30% anatase:rutile with a small amount of amorphous phase): surface area of 55 ± 15 m^2^·g^−1^ [28] and particle size of ∼30 nm [29]. Cobalt (II) sulfate heptahydrate (CoSO_4_·7H_2_O, ≥97%) was from Aldrich, and phenol (C_6_H_5_OH, 99.5%) from Sigma. Acetonitrile (HPLC grade) was from J.T. Baker. O_2_ gas (purity ≥ 99.995%) was used in some experiments. All chemicals were used without further purification. Distilled water was obtained from a Millipore apparatus (Milli-Q water), resistivity of 18.2 MΩ at 298.0 K and total organic carbon (TOC) less than 5 μg·L^−1^.

### 2.2. Catalyst Synthesis

Wetness impregnation was used to immobilize Co(II) on the surface of TiO_2_ nanoparticles to prepare Co/TiO_2_ photocatalysts [30] with enhanced photocatalytic properties. CoSO_4_·7H_2_O was dissolved in distilled water and impregnated on Degussa TiO_2_-P25 nanoparticles under vigorous stirring.

This suspension was stirred for 24 h, and then dried in an oven at 50 °C to dry it completely. Then, the photocatalysts were calcined in a furnace (Paragon model HT-22D, Thermcraft) at 600 °C for 4 h with a ramp rate of 10 °C·min^−1^. Finally, the so-prepared catalysts were ground thoroughly and labeled as Co(X%)/TiO_2_, where X denotes the Co(II) mass percent relative TiO_2_, X = 0.1%, 0.3%, 0.5%, and 1%. TiO_2_ particles turned from white to yellowish as the cobalt percentage increased.

The samples were placed in water under stirring for 2 h to test for potential Co^n+^ leaching; after filtration, the solution was analyzed using ICP/MS and the dry solid by XRD. No evidence of Co^n+^ leaching was found (Table 1).

### 2.3. Characterization

X-ray diffraction (XRD) measurements were conducted with a Bruker Siemens D5000 diffractometer following Bragg–Brentano geometry and θ/2θ configuration and equipped with a graphite monochromator. The optics consist of primary and secondary 2° Soller slots, a variable output slot, a 1mm receiver slot, a 0.2 mm monochromator slot, and a 0.6 mm detector slot. The detector was a scintillation counter. The acquisition conditions were a sweep range (2θ): 2–80°, a jump size (step): 0.050°, and the acquisition time at each jump was about 2.5 s (time per jump). The DiffracPlus software v. 8.0.0.2 (Socabim) was used for data processing. 

The morphology of TiO_2_-P25 and Co(X%)/TiO_2_ were studied using a Jeol JSM-IT100 scanning electron microscope (SEM) equipped with an energy dispersive X-ray (EDX) system to obtain information on the surface elemental composition. The samples were deposited on thin films of amorphous carbon and coated with gold (2 nm). Transmission electron microscopy (TEM), using a Jeol JEM 1100 instrument, operating at an acceleration voltage of 80 kV, was also employed to examine the photocatalysts’ surface morphology. Samples were prepared by depositing a few drops of nanoparticle suspension on carbon-coated copper grids (electron microscopy, 200 mesh) and air drying.

Textural characterization was carried out using adsorption/desorption isotherms of N_2_, as adsorption gas, at 77.4 K (Tristar II Plus—Micromeritics, automatic station with 3 simultaneous measurement ports). Helium was used for the measurement of the dead volume of the sample holders. Isotherms were measured in the range P/P_0_ = 0.1–1.0. The range of the BET area used for the calculation of the specific surface area was P/P_0_ = 0.05–0.3. “Microactive for Tristar II Plus”, v.2.03 (Micromeritics), was the software for control, acquisition, and data processing.

Raman measurements of the dried samples on glass were performed using a Witec Alpha 300 R confocal Raman system equipped with 633 and 785 nm excitation laser lines and a grating of 600 gr·cm^−1^. Raman spectra were acquired at room temperature using a 20× objective over the spectra range of 90–2500 and 90–1800 cm^−1^ for 633 and 785 nm, respectively, laser powers at the samples of 21 mW (633 nm), 28 and 35 mW (785 nm), and acquisition times in the range of 0.5–5 s with 10 accumulation. The Raman spectra were processed with the Project5.2 Witec software for peak assignment including position, height, and FWHM. SpectraGryph 1.2 software was used for spectral processing involving baseline, background removal (i.e., Raman spectrum of glass was subtracted for the spectra acquired using a 785 nm laser line), and cosmic ray removal correction. Raman shift calibration was performed using a silicon wafer (peak at 520 cm^−1^). The spectra were normalized to the E_g_ peak (146 cm^−1^) for a better comparison between samples.

Co(0.1%)/TiO_2_ photocatalyst was analyzed by X-ray photoelectron spectroscopy (XPS) using a Thermo Scientific K-Alpha ESCA instrument equipped with an Al Kα monochromatized radiation (hν = 1486.7 eV) X-ray source. Measurements were carried out in a Constant Analyzer Energy mode with a 100 eV pass energy for survey spectra, and 20 eV pass energy for high-resolution spectra. The spot size selected was 400 μm and K-Alpha’s charge compensation system was employed during analysis. In data analysis, C 1s = 285.0 eV was taken as the binding energy reference. Surface elemental composition was determined using the standard Scofield photoemission cross sections.

The UV-Vis diffuse reflectance spectra (DRS) (200–800 nm) of the solid photocatalyst was measured on a JASCO V-560 UV-Vis spectrophotometer with a double monochromator and double beam optical system, equipped with an integrating sphere attachment (JASCO ISV-469, Oklahoma City, OK, USA). Reflectance spectra were converted to equivalent absorption Kubelka–Munk units.

### 2.4. Photocatalytic Activity

The photocatalytic activity of the synthetized samples was tested in an axial photoreactor, under UV and near-UV-Vis light (NUV-Vis), monitoring changes in phenol (C_6_H_5_OH) concentration in aqueous solution. Two types of photocatalytic experiments were carried out. The first was performed under NUV-Vis light, in which the photoreactor was equipped with a Heraeus TQ 150 medium-pressure Hg-vapor lamp, with intense emission lines at 254, 313, 366, 405, 436, 546, and 578 nm. A Duran 50^®^ glass jacket, filled with water, was used to cut off the radiation below 366 nm. For the second experiment, the photoreactor was equipped with a Heraeus TNN 15/32 low-pressure Hg-vapor lamp, with a single intense emission line at 254 nm, located inside a quartz tube. The photon flux at 366 nm, as determined by potassium ferrioxalate actinometry, [31] was 2.38 × 10^−6^ Einstein·s^−1^, and at 254 nm, it was of 8.33 × 10^−8^ Einstein·s^−1^.

The photocatalytic reactions were performed under a coherent magnetic stirring of 200 mL phenol solution, with an initial concentration of 50 mg·L^−1^ to facilitate monitoring, mixed with 200 mg of photocatalyst under controlled O_2_ pressure. All samples were kept in the dark for 30 min, to ensure adsorption–desorption equilibrium. The heterogeneous suspensions, at natural pH, were exposed to UV irradiation for 60 min and to NUV-Vis irradiation for 300 min at ca. 25 °C, maintained by water flow from a thermostat-cryostat. Samples of aqueous phenol and photocatalysts were taken at time intervals and filtered through Sartorius NY 0.45 ™ filters.

Phenol concentration was monitored using a Biochrom Libra S70 spectrophotometer to measure the UV-Vis absorbance at 270 nm and using HPLC UV-Vis at 210 and 270 nm, in a Thermo Fisher instrument equipped with a 6000 LP UV detector, an AS 3000 autosampler, and a P4000 solvent pump. A KROMAPHASE C18 column (4.6 mm × 150 mm × 5 μm) was used, with a volume of 50 μL injected, a flow rate of 1.0 mL·min^−1^, at 30 °C, the mobile phase being acetonitrile and water (25:75, *v*/*v*). Replicate experiments showed differences in rate constants within 5% error.

Total organic carbon (TOC) removal was measured using a ShimadzuTOC-5000A analyzer. Photoproducts were identified using HPLC/MS (Thermo Scientific LTQ Orbitrap Discovery), equipped with an electrospray interface operating in negative ion mode (ESI-), with a Phenomenex Kinetex XB-C18 column (100 mm × 2.6 μm) operating at 30 °C with elution solvents A (0.1% formic acid) and C (0.1% methanol) at a flow rate of 200 μL·min^−1^. The gradient followed was 0–1 min, 95–95% of A and 5–5% of C; 1–8 min, 95–5% A and 5–95% C; 8–10 min, 5–5% A and 95–95% C; 10–11 min, 5–95% of A and 95–5% C; and 11–15 min, 95–95% A and 5–5% C. The amount injected was 5–25 μL. The analyses were carried out using full-scan data-dependent MS scanning from *m*/*z* 50 to 500. The obtained photoproducts were the same found in the photocatalyzed degradation of phenol using surface-impregnated TiO_2_ (M = Cu, Cr or V) as photocatalysts [32].

## 3. Results and Discussion

### 3.1. Characterization of the Catalysts

#### 3.1.1. X-ray Diffraction

**Figure 1:**
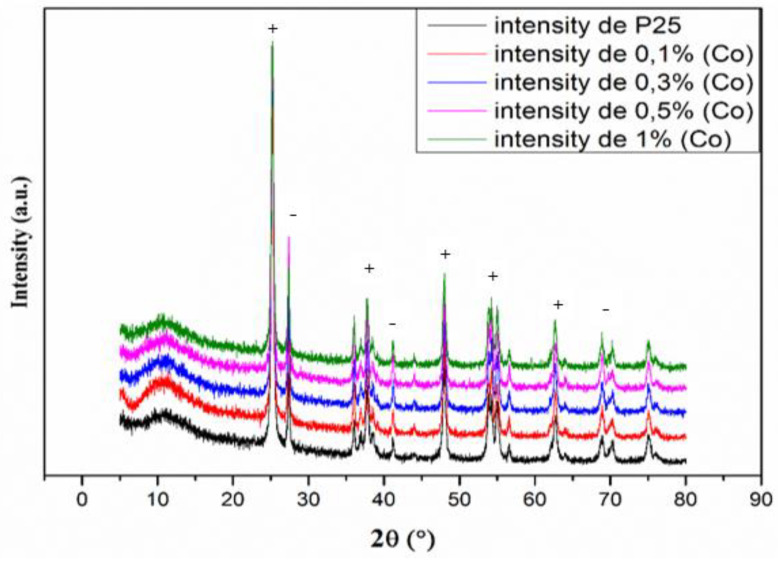
X-ray diffraction patterns of pure TiO_2_-P25 and Co/TiO_2_ samples with different Co/Ti ratio (+: anatase, −: rutile).

The crystal structure of the synthesized samples was evaluated using XRD analysis, and the results are shown in Figure 1. Pure TiO_2_-P25 shows eight primary peaks at 25.20°, 36.52°, 37.78°, 48.00°, 53.96°, 56.86°, 62.74°, and 75.08°, which are assigned to anatase diffraction planes: (1 0 1), (1 0 3), (0 0 4), (2 0 0), (1 0 5), (2 1 1), (2 0 4), and (2 1 5). The four peaks located at 27.34°, 36.02°, 41.14°, and 55.00° can be attributed to rutile diffraction planes: (1 1 0), (1 0 1), (1 1 1), and (2 1 1) [33]. The diffraction patterns of Co(0.1%)/TiO_2_, Co(0.3%)/TiO_2_, Co(0.5%)/TiO_2_, and Co(1%)/TiO_2_ photocatalysts were very similar to that of nonimpregnated TiO_2_-P25. No other metal and/or metal oxides were observed, which could be related to the low Co content (0.1%, 0.3%, 0.5%, and 1% by weight) and its uniform distribution onto the TiO_2_ surface [30]. Table 2 shows the crystalline phase percentage of anatase and rutile calculated from their most intense reflections (1 0 1) and (1 1 0), respectively, using the Spurr and Myers equation [34]. The anatase to rutile ratio decreases when going from P25 [35] to Co(X%)/TiO_2_ due to the dopant Co^2+^ promoting anatase to rutile phase transformation [16].

The substitution of Ti^4+^ by Co^2+^, with slightly different size, induces a charge imbalance which implies the creation of oxygen vacancies to maintain charge neutrality [36]. Co^2+^ and oxygen vacancies introduce defect levels acting as trapping sites, therefore diminishing electron–hole recombination [37]. Effect levels imply the presence of interband states which means a reduction in the band gap, boosting the photocatalytic activity under Vis radiation.

Crystallite size was calculated using the Scherrer equation [38]. The observed trend with both polymorphs is to increase in size continuously from TiO_2_-P25 to the doped photocatalyst, with a tendency to reduce in size as the doping percentage increases. A similar behavior has been reported for rutile [39]. Doping, within the used percentages, does not affect cell parameters (Table 2), which is consistent with the substitution of Ti^4+^ by Co^2+^ in the doped photocatalyst, provided they show relatively similar radii (74.5 vs. 79 pm, respectively, considering low spin Co^2+^ and 6-coordination) [40]. The fact that cell parameters are not affected is in agreement with doping taking place mainly on the surface and not on the bulk.

As stated above, 1 g of a Co(0.1%)/TiO_2_ sample in 1 L of distilled water was stirred for 2 h. The solid recovered after filtration and drying was XRD analyzed. The same diffraction peaks of Co(0.1%)/TiO_2_ were found before and after the test (Figure S1 of Supplementary Materials); therefore, similar values were obtained for the derived parameters, thus confirming the stability of the as-prepared photocatalyst.

#### 3.1.2. Raman Spectroscopy

**Figure 2:**
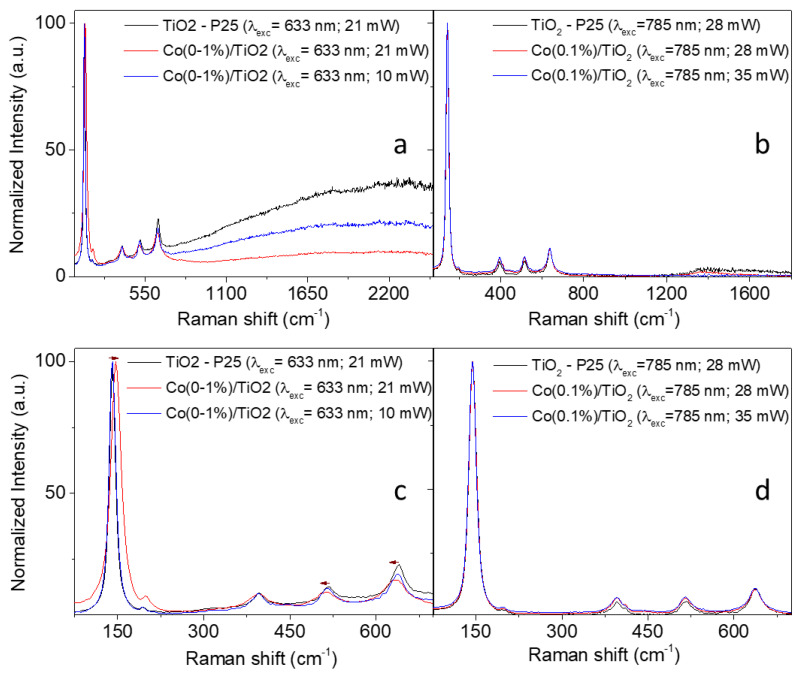
Raman spectra of Co(0.1%)/TiO_2_ photocatalyst acquired using (**a**) 633 nm and (**b**) 785 nm excitation lasers. Two different laser powers were used: 21 and 10 mW for 633 nm and 28 and 35 mW for 785 nm. Spectra shown in (**c**,**d**) are centered in a smaller spectra window (90 to 670 cm^−1^) for a better observation of the Raman peaks corresponding to the anatase phase.

Figure 2 shows the Raman spectra of Co(0.1%)/TiO_2_, which are compared with the Raman spectrum of undoped TiO_2_, acquired using two different lasers, 633 and 785 nm, and two laser powers. Anatase polymorph, a tetragonal structure, is present. The Raman active “lattice vibrations” shown in Table 3 are assigned based on the D_4h_ point group.

The very low intensity band at ca. 445 cm^−1^, E_g_ mode of the rutile phase, does not allow a rough estimation of the weight ratios of anatase phase to rutile phase from the Raman spectrum. The table also includes Ti-O and Ti-Ti bond lengths calculated using covalence/length/frequency relations [41]. Values obtained for Co(0.1%)/TiO_2_ are similar to those reported for P25, which agrees with the tiny structural effect of 0.1% Co doping observed using XRD.

The method of preparation of the doped photocatalysts determines its particle size, which has to do with oxygen vacancies and electron–phonon coupling. Both factors may influence shifts and broadening of Raman peaks. Contrary to Co-doped TiO_2_ synthetized using sol-gel [42], the as-prepared photocatalyst does not show a wavenumber shift upon the excitation of 785 nm laser line at any of the power laser values used. The FWHW of the most intense peak is the same for the undoped and doped (Co(0.1%)/TiO_2_) sample (Figure 2d, Table S1), the corresponding phonon lifetime being 0.34 ps. Interestingly, a red shift of 6 cm^−1^ was observed in the peak centered at 141 cm^−1^, while the peaks centered at 517 and 640 cm^−1^ displayed a blue shift of 4–5 cm^−1^ (Figure 2c and Table S1 for peak at 141 cm^−1^) upon the excitation of 633 nm with a laser power of 21 mW. However, these shifts were not observed at lower powers of the 633 nm laser (Figure 2c,d, Table S1 for peak at 141 cm^−1^). This laser-induced phase transition (*e.g.*, low crystallinity or phase transformation) in Co-doped anatase TiO_2_ nanoparticles was previously reported [43]. This laser power dependence was not observed with the 785 nm laser line since its energy is lower than that of the 633 nm one.

Figure S2 shows that the intensity of the well-defined peaks is higher for the Co-doped photocatalyst using both laser lines (see Table S1 for the most intense peak in Supplementary Materials), such enhancement has been previously described [44,45].

#### 3.1.3. Scanning Electron Microscopy and Energy Dispersive Spectroscopy

**Figure 3:**
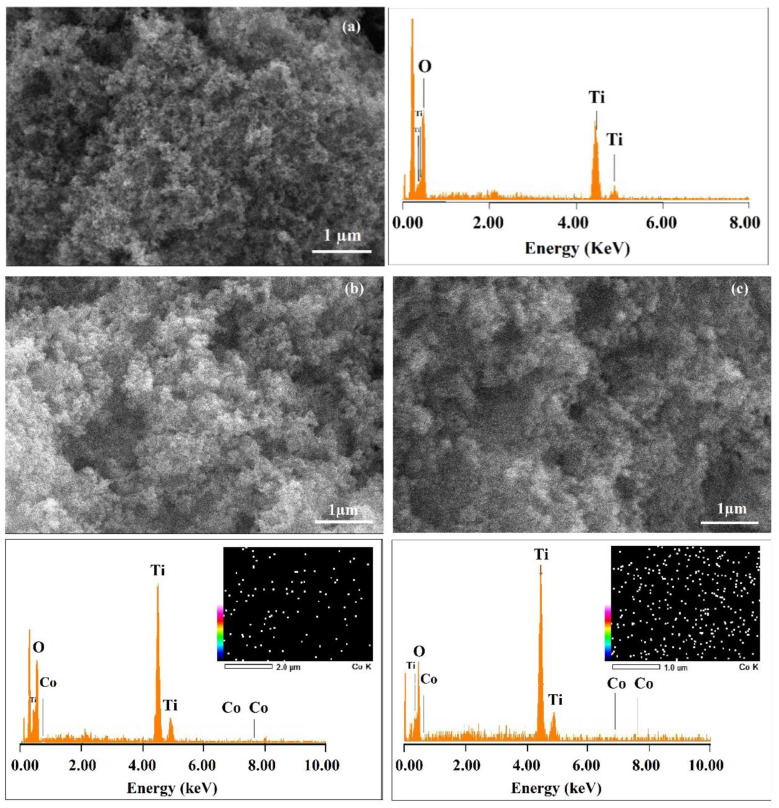

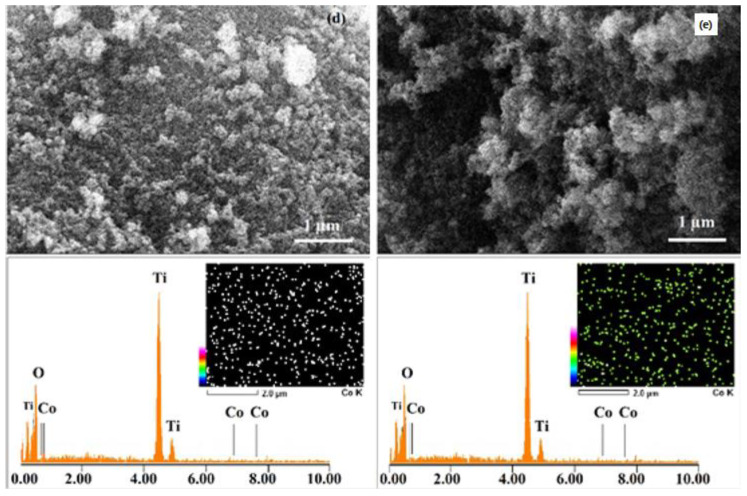
SEM/EDS micrographs of (**a**) TiO_2_-P25 and Co(X%)/TiO_2_ samples as (**b**) Co(0.1%)/TiO_2_, (**c**) Co(0.3%)/TiO_2_, (**d**) Co(0.5%)/TiO_2_, (**e**) Co(0.5%)/TiO_2_.

The morphology and elemental analysis of the Co(X%)/TiO_2_ samples were studied using scanning electron microscopy/energy dispersion spectroscopy (SEM/EDS). SEM images, Figure 3, show the crystals’ shape and size of all cobalt-loaded samples are similar to those of unmodified P25-TiO_2_ [17], with an average crystal size of 30 nm, in agreement with the value obtained from XRD data (vide supra). EDS analysis shows the percent of cobalt matches the initial doped amount at the lowest percentages, while at higher Co/TiO_2_ doping percentages, the presence of Co is lower on the surface (Table 4), which could be attributed to Co ions diffusing into inner layers of the photocatalyst, a process that is facilitated by oxygen vacancies. The EDS-mapping images of the doped photocatalysts’ surfaces revealed a homogeneous distribution of cobalt ions [35]. Considering typical penetrations of X-rays in SEM/EDS, the Co ions reached depths of not less than 2 μm below the surface. The presence of Co with an optimal concentration (0.1%) prevents the TiO_2_-P25 particles from agglomerating, probably due to the development of a surface charge. Large aggregates can decrease photocatalytic activity by reducing the exposed surface and shading active sites from exciting radiation [23].

#### 3.1.4. XPS Analysis

**Figure 4:**
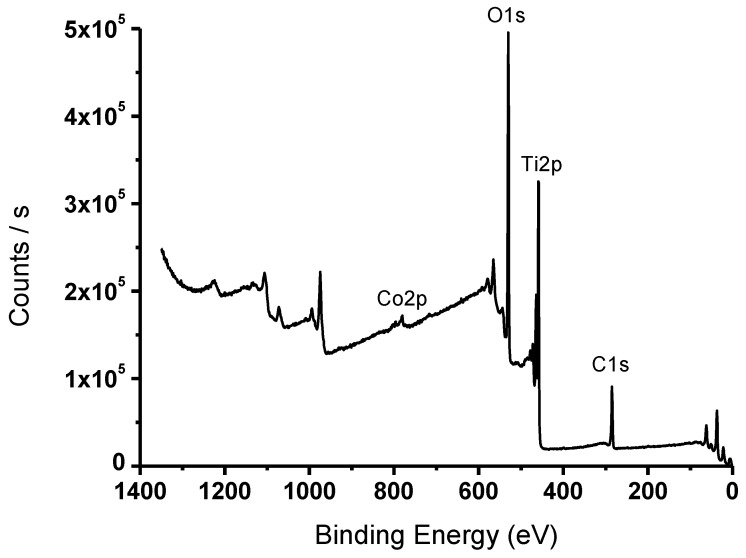
Survey XPS spectrum of the Co(X%)/TiO_2_ photocatalyst.

Co(X%)/TiO_2_ samples were analyzed using XPS, in an attempt to understand the bonding states of the surface elements on the doped photocatalyst. The survey XPS spectrum is shown in Figure 4. The atomic composition of C 1s, Ti 2p, O 1s, and Co 2p of the Co/TiO_2_ photocatalyst was 27.8%, 20.1%, 50.3%, and 1.8%, respectively. The characteristic satellite (shake up) of Co at around 786 eV is evidence of the presence of Co(II). The high-resolution Co 2p spectrum showed spin-orbit splitting into 2p_1/2_ and 2p_3/2_ components (Figure S2a). The Co 2p spectrum is close, or quite similar, to the Co(OH)_2_ compound [46]. The dominant Ti 2p_3/2_ peak is located at 458.8 eV binding energy and Ti 2p_1/2_ at 464.5 eV, i.e., with a spin-orbit splitting value of 5.7 eV, corresponding to Ti(IV) [47], Figure S2b. XPS Ti 2p peaks appear at slightly higher binding energy than in the case of P25 (458.6 and 464.2 eV, respectively). Electron density is transferred from Ti(IV) to Co(II), due to the higher Pauling electronegativity of the latter, and the binding energy increases. The peak at ca. 530 eV (O 1s, Figure S2c) is characteristic of the Ti–O lattice bond of TiO_2_ [48].

#### 3.1.5. Textural Properties

**Figure 5:**
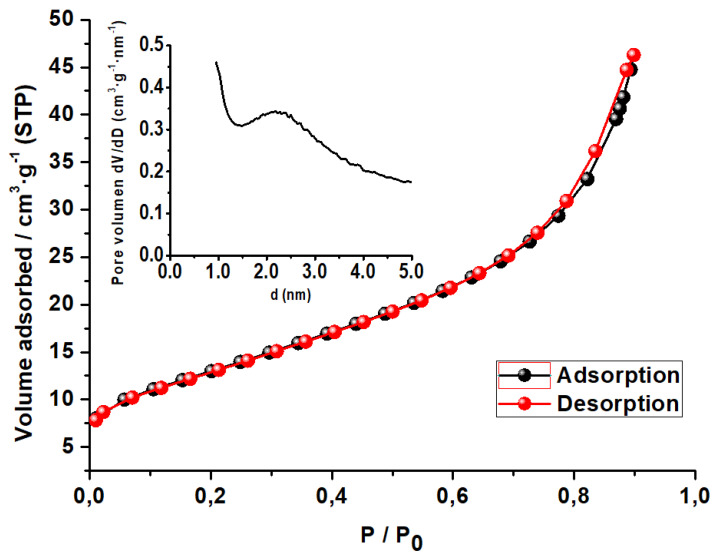
N_2_ adsorption–desorption isotherm of Co(0.1%)/TiO_2_ photocatalyst. Inset: pore size distribution.

The N_2_ adsorption–desorption isotherm of Co(0.1%)/TiO_2_ is displayed in Figure 5, which according to the IUPAC classification belongs to type IV with a very small hysteresis loop H3 (ca. 0.75–0.9 P/P_0_), suggesting the mesoporous nature of the photocatalyst [49]. The adsorption isotherm does not level off at high pressure, which implies the existence of slit-shaped pores formed by nonrigid aggregate particles and no pore networks according to BJH [50].

The BET isotherm was used to calculate the surface area (SBET—multipoint), and the textural properties were analyzed based on the Barrett, Joyner, and Halenda (BJH) model [50] (Table 5). The observed texture is different from that of Co(0.1%/TiO_2_) as prepared by the hydrothermal method, where the isotherm, also type IV, shows a large H3 hysteresis loop (0.4–0.8 P/P_0_), and the reported values of SBET (22.8 m^2^·g^−1^) and pore volume (0.033 cm^3^·g^−1^) are smaller than those of the as-synthesized photocatalyst [51]. Pore size distribution shows a maximum at 22 Å, just in the lower mesopore limit.

#### 3.1.6. UV-Vis Diffuse Reflectance Spectroscopy

**Figure 6:**
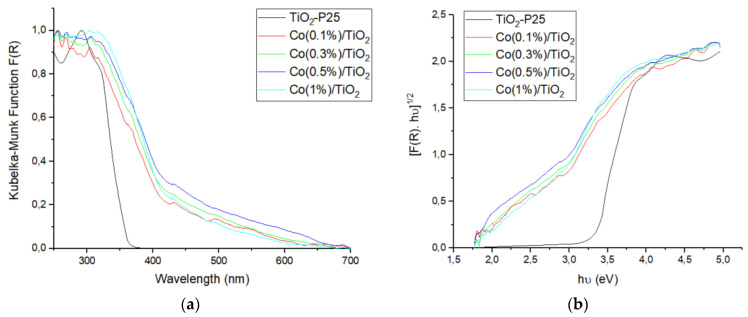
UV-Vis diffuse reflectance spectra of pure TiO_2_-P25 and Co/TiO_2_ samples (**a**) Kubelka–Munk; (**b**) Tauc plot (indirect band gap).

The UV-Vis DRS spectra of TiO_2_-P25 and the prepared photocatalysts are shown in Figure 6a. The absorption band of TiO_2_-P25, occurring from 200 to 380 nm, is associated with the O^2−^ (2p) → Ti(IV) (3d) transitions and tetrahedral symmetry [52]. In the case of Co(II)-doped TiO_2_ samples, band tailing appears at ca. 400 nm; such light absorption increase in the Vis region, as compared to pure TiO_2_-P25, relates to the charge transfer from O^2−^ to Co(II) [53] and to the Vis absorption of the electrons from the defect states, the intensity being directly related to the number of defect states [54]. Visible light absorption increases as less energy is required to move electrons to the conduction band. The band gap (E_g_) was obtained using the Tauc plot, Figure 6b [55]. For pure TiO_2_-P25, the band gap was found equal to 3.3 eV in agreement with the literature [56]. For cobalt-doped TiO_2_ photocatalysts, E_g_ narrows and depends on Co concentration (Table 6), with values around 2.3–2.4 eV. Table S2 lists the band gap of several Co-doped TiO_2_ photocatalysts. It follows that the band gap depends on the synthetic method and on the nature of the starting material. The E_g_ narrowing, observed as an absorption onset shift to the visible region, is due both to the substitution of Ti(IV) by Co(II), which implies new d-states coming from Co- in the forbidden band of TiO_2_, and to the addition of energy levels in the same zone from oxygen vacancies [36].

The presence of cobalt energy levels over the Fermi level enables visible light absorption and prevents e^−^/h^+^ pair recombination, thus enhancing the photocatalytic efficiency, but an excess of dopant facilitates hole–electron pair recombination, reducing the photocatalytic activity [36,37].

### 3.2. Kinetics of Phenol Photodegradation

The photocatalytic activity of TiO_2_ and Co-TiO_2_-doped samples was tested by monitoring phenol photodegradation upon UV and NUV-Vis radiation. After irradiation, e^−^/h^+^ pairs are formed according to the usual scheme of photocatalysis with semiconductors, [57,58] and the dopant Co(II) traps e^−^, thus reducing the probability of recombination. O_2_ and H_2_O/HO^−^ adsorbed onto the photocatalyst surface scavenge the trapped e^−^ and h^+^ to form reactive oxygen species (hydroxyl, HO^•^, hydroperoxy, HO_2_^•^, and superoxide, O_2_^•−^, radicals, and H_2_O_2_), which are involved in the oxidative photodegradation of phenol, same as h^+^.

**Figure 7:**
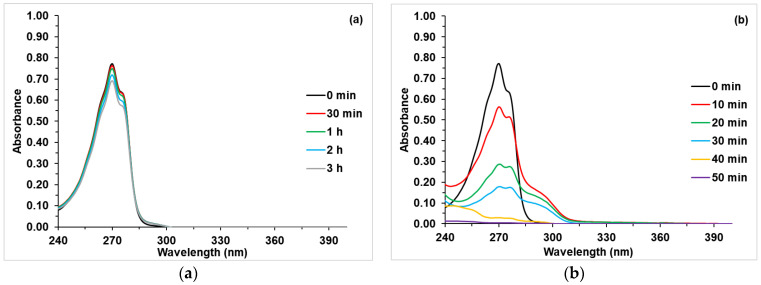
Photocatalytic activity of P25-TiO_2_ in phenol degradation under (**a**) visible and (**b**) UV irradiation. [Phenol] = 50 mg·L^−1^; P25-TiO_2_ = 1g·L^−1^, T ca. 25 °C, natural pH.

In the case of pure TiO_2_, the degradation of phenol was found to be effective under UV light; which was not the case under NUV-Vis light (Figure 7). The same results were found for the photodegradation under NUV-Vis light, of dyes [18,59], drugs [60], and phenol [19]. In fact, the large band gap of TiO_2_ makes the absorption of visible light photons ineffective, minimizing its photoactivity [61].

**Figure 8:**
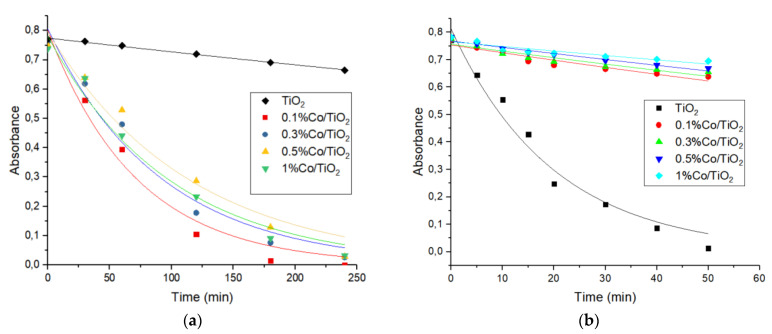
Absorbance vs. time curves for phenol degradation under (**a**) NUV-Vis and (**b**) UV irradiation using TiO_2_-P25 and Co(X%)/TiO_2_ photocatalysts. [Phenol] = 50 mg·L^−1^; Photocatalyst = 1g·L^−1^, T ca. 25 °C, natural pH.

In the case of Co(X%)/TiO_2_ photocatalysts, phenol photodegradation was found to be totally different from pure P25-TiO_2_ (Figures S4 and S5). Figure 8 shows the absorbance vs. time curves for phenol photodegradation in the presence of TiO_2_-P25 and Co(%)/TiO_2_ photocatalysts. The as-prepared photocatalysts slightly degrade phenol under UV light, whereas complete phenol photodegradation was observed under Vis light. Table 6 summarizes the obtained pseudo first order rate constants. Although, under UV irradiation, the rate constants are higher with Co-doped TiO_2_, there is only a very small phenol disappearance; a similar behavior was observed with Cu-doped titanium [32]. On the other hand, under NUV-Vis radiation, the rate constant increases when the band gap decreases, which runs parallel to Co content.

The photocatalytic effect was more important with the lowest concentration of doped metal, i.e., Co(0.1%)/TiO_2_. As stated above, the fastest phenol photodegradation happen with the Co(0.1%)/TiO_2_ photocatalyst, probably due to a better dispersion of metal ions on the TiO_2_-P25 surface (as found by SEM, Section 3.1.3). The same results were found by previous authors with Cu-doped titania [62]. At higher Co concentrations, the rate constants decreased slowly; in agreement with the well-known fact that above the optimal value the presence of dopant reduces the photoactivity, charges are trapped and/or recombination centers are formed [25].

### 3.3. Total Organic Carbon (TOC) Analysis

**Figure 9:**
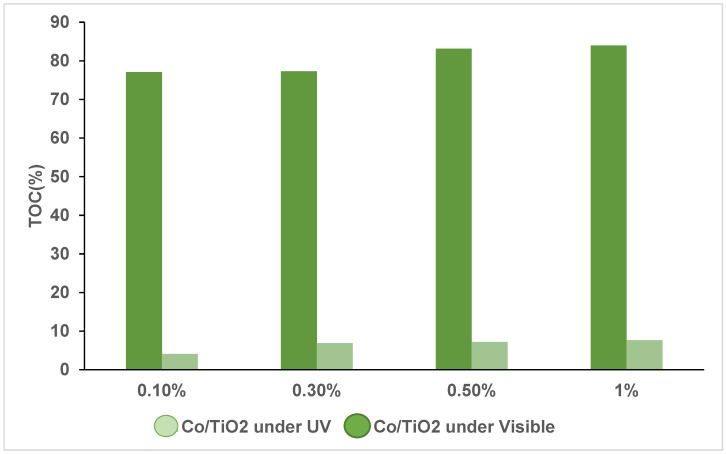
TOC percentage removal for phenol photodegradation using Co(X%)/TiO_2_ photocatalysts. Irradiation time: NUV-Vis 240 min; UV 60 min. [Phenol] = 50 mg·L^−1^; Photocatalysts = 1g·L^−1^, T ca. 25 °C, natural pH.

To determine the degree of mineralization during photocatalysis, TOC analyses were carried out after 60 min of photoirradiation under UV light and 240 min under NUV-Vis light (Figure 9). The initial phenol concentration of 50 mg·L^−1^ with a measured TOC value of 48 mg·L^−1^ was used.

TOC values decreased drastically upon photocatalysis, from 48 to 1.97 mg·L^−1^ under NUV-Vis light in the presence of Co(X%)/TiO_2_ photocatalysts, i.e., a 96% TOC removal, whereas the observed removal was much lower (23%) under UV light. For both irradiation conditions, the maximum TOC removal was observed for the lowest dopant content (Co(0.1%)/TiO_2_), and in accordance with kinetics results, it decreased as the Co percent was increased.

TOC results are in agreement with kinetic results; the observed absorbance decay follows the same pattern found for TOC, irrespective of the irradiation wavelength: small decay and TOC reduction with UV and almost complete elimination with NUV-Vis.

### 3.4. Photodegradation and Energetic Efficiency of the Process

Using the light intensity (I_λ_/Einstein·L^–1^·s^–1^) at the irradiation wavelength (λ), the molar absorptivity at λ (ε_λ_/mol·dm^–3^·cm^–1^), the pathlength of the photoreactor (l/cm), and the apparent pseudo first order rate constant (k/s^–1^), the photocatalysis quantum yield (Φ_photodegradation_) can be calculated (Table 7) [28]; likewise, taking into account the apparent pseudo first order rate constant (k/s^–1^), the electric power consumed by the lamp (P/kW), and the solution volume (V/L), the energy efficiency parameter (E_EO_/kW·L ^–1^·s ^–1^), i.e., the energy required to reduce the pollutant concentration per volume and time unit, can be obtained [32] (Table 7).

The large value of the UV photodegradation quantum yield suggests the contribution of secondary processes, possibly chain processes. On the other hand, EEO is much more favorable with UV radiation. At first glance, the choice would be to use UV radiation if the irradiation source is to be solely powered by electric current. The advantage of using the prepared photocatalyst, under NUV-Vis, is a much higher level of TOC removal and the use of sunlight, which would make the process more sustainable and reduce energy-associated costs.

As mentioned above (vide supra), the catalyst can be washed with no lixiviation or structural consequences after use, and when reutilized, it showed similar kinetic and TOC reduction results.

## 4. Conclusions

The degradation of phenol was investigated using cobalt-impregnated TiO_2_ samples with different concentration, Co(X%)/TiO_2_, with X = 0.1%, 0.3%, 0.5%, and 1.0%, under NUV-Vis (>366 nm) and UV (254 nm) radiation. The TiO_2_-P25 surface was impregnated, and the obtained solids were characterized by XRD, Raman, XPS, SEM, EDS, and TEM. Crystallite size increased in the doped samples, where Ti(IV) was substituted by Co(II) and oxygen vacancies were created as well as interband states, which reduced the band gap as found with UV-Vis DRS measurements. The N_2_ adsorption isotherm corresponds to type IV with a small H3 loop near the maximum relative pressure. The photocatalytic activity over phenol photodegradation was found to be the best for Co(0.1%)/TiO_2,_ at the lowest found band gap (E_g_ ca. 2.3–2.4 eV)_,_ when using NUV-Vis radiation. The TOC test showed an approximately complete removal (96%) for phenol photodegradation under NUV-Vis radiation and Co(0.1%)/TiO_2_, while it only removed 23% under UV radiation.

In summary, we easily prepared, by impregnation, and characterized a Co- doped TiO_2_ (P25) photocatalyst showing extended light harvesting into the Vis region and were able to completely photodegrade phenol in an aqueous solution (50 ppm) after 3 h under NUV-Vis irradiation. The so-prepared catalyst was found to be chemically and mechanically stable and showed similar kinetic and TOC results upon reutilization.

## Figures and Tables

**Table 1:** Co(II) percent in Co/TiO_2_ catalysts after 2 h stirring in water.

Starting material	0.1000%	0.3000%	0.5000%	1.0000%
After 2 h	0.0995%	0.2950%	0.5053%	1.0180%

**Table 2:** Crystalline phase percentage, anatase and rutile main reflections (2θ^0^), crystallite size (Scherrer), and unit cell parameters (tetragonal) from XRD analysis of P25 and Co(X%)/TiO_2_.

	Parameter	P25	Co(0.1%)	Co(0.1%) ^a^	Co(0.3%)	Co(0.5%)	Co(1.0%)
Anatase	wt%	75	69	70	68	63.5	71
	Crystallite size (nm)	21.99	26.37	25.24	25.05	26.93	24.70
	a = b (nm)	0.3800	0.3796	0.3770	0.3769	0.3796	0.3795
	c (nm)	0.9449	0.9490	0.9522	0.9530	0.9473	0.9486
Rutile	wt%	25	31	30	32	36.5	29
	Crystallite size (nm)	31.43	36.58	34.20	38.96	30.13	32.40
	a = b (nm)	0.4610	0.4606	0.4603	0.4583	0.4603	0.4603
	c (nm)	0.2962	0.2961	0.2956	0.2952	0.2966	0.2963

^a^ After stirring in water for two hours.

**Table 3:** Raman active “lattice vibrations” for anatase in P25 [38] and in Co(0.1%)/TiO_2_, Ti-Ti bond lengths (octahedral chain) and Ti-O bond lengths.

Mode	Photocatalyst	Vibration Wavenumber (cm^−1^) and Bond Length (Å)		
E_g_	P25	143 (d_Ti-Ti_: 2.85)	196 (d_Ti-Ti_: 2.65)	638 (d_Ti-O_: 2*x* 1.89)
	P25 (this work)	144 (d_Ti-Ti_: 2.85)	198 (d_Ti-Ti_: 2.64)	638 (d_Ti-O_: 2*x* 1.89)
	Co(0.1%)/TiO_2_	144 (d_Ti-Ti_: 2.85)	197 (d_Ti-Ti_: 2.65)	637 (d_Ti-O_: 2*x* 1.89)
B_1g_	P25	396 (d_Ti-O_: 3*x* 2.20)		
	P25 (this work)	396 (d_Ti-O_: 3*x* 2.20)		
	Co(0.1%)/TiO_2_	395 (d_Ti-O_: 3*x* 2.20)		
A_1g_	P25	515 (d_Ti-O_: 3*x* 2.03)		
	P25 (this work)	518 (d_Ti-O_: 3*x* 2.02)		
	Co(0.1%)/TiO_2_	515 (d_Ti-O_: 3*x* 2.03)		

**Table 4:** Cobalt weight percent content for Co(X%)-TiO_2_ samples by EDS analysis.

Sample	Co(0.1%)/TiO_2_	Co(0.3%)/TiO_2_	Co(0.5%)/TiO_2_	Co(1%)/TiO_2_
Co (Weight %)	0.11	0.29	0.46	0.69

**Table 5:** Textural properties of Co(0.1%)/TiO_2_.

Photocatalyst		Co(0.1%)/TiO_2_		
BET	S_BET_/m^2^·g^−1^	39.58 ± 0.07		
	Constant C	102.83		
	V_m_ (monolayer adsorption volume)/cm^3^·g^−1^		9.09	
Parameter		Surface area (m^2^·g^−1^)	Pore volume (cm^3^·g^−1^)	Average pore width (4V/S Å)
t-plot external surface area		39.50		
t-plot micropore volume			−0.000284	
BJH adsorption		36.815 ^a^	0.055445 ^b^	60.241
BJH desorption		37.0671 ^a^	0.055555 ^b^	59.950
D-H adsorption		36.726 ^a^		60.238
D-H desorption		36.9780 ^a^		59.943
*Maximum pore volume* at p/p°/cm^3^/g (STP)			0.176986	Median pore width
			0.016283	7.736
Average particle size/Å		1515		

^a^ Cumulative surface area of pores between 1.7 and 300 nm in diameter. ^b^ Cumulative pore volume of pores between 1.7 and 300 nm in diameter.

**Table 6:** Band gap of TiO_2_-P25 and Co-doped TiO_2_ samples and pseudo first order rate constants for phenol degradation under NUV-Vis and UV irradiation. [Phenol] = 50 mg·L^−1^; Photocatalyst = 1g·L^−1^, T ca. 25 °C, natural pH.

Photocatalyst	Indirect Eg (eV)	Vis		UV	
		k·10^2^ (min^−1^)	R^2^	k·10^2^ (min^−1^)	R^2^
TiO_2_-P25	3.3	0.060 ± 0.002	0.90	3.6 ± 0.8	0.97
0.1% Co/TiO_2_	2.4	1.1 ± 0.2	0.98	4.5 ± 0.7	0.96
0.3% Co/TiO_2_	2.3	0.8 ± 0.2	0.98	5.2 ± 0.3	0.90
0.5% Co/TiO_2_	2.3	0.41 ± 0.09	0.96	3.8 ± 0.5	0.96
1.0% Co/TiO_2_	2.3	0.7 ± 0.2	0.98	5.5 ± 0.9	0.86

**Table 7:** Photodegradation quantum yield (Φ_photodegradation_) and energy efficiency (E_EO_) for phenol photocatalyzed degradation over Co(0.1%)/TiO_2_ under UV and NUV-Vis irradiation.

Irradiation Source	UV	NUV-Vis
Φ_photodegradation_	9.77	0.67
E_EO_/kW·L ^–1^·s ^–1^	766	156835

## Supplementary Materials

The following supporting information can be downloaded at: https://www.mdpi.com/article/10.3390/ma16114134/s1, Figure S1. Diffractograms of Co(0.1%)/TiO_2_, a: before leaching test, b: after leaching test. Figure S2: Raman spectra of TiO_2_-P25 and Co(0.1%)/TiO_2_ acquired using (a) 633 nm and (b) 785 nm excitation laser line. Figure S3. XPS spectra (a) Co 2p scan and fitting; (b) Ti 2p scan; (c) O 1s scan; (d) C 1s scan. Figure S4: Time-resolved UV-Vis spectra of phenol during its UV irradiation in the presence of (a) Co(0.1%)/TiO_2_, (b) Co(0.3%)/TiO_2_, (c) Co(0.5%)/TiO_2_, (d) Co(1%)/TiO_2_. [Phenol] = 50 ppm; Photocatalysts = 1g·L^−1^, T ca. 25 °C, natural pH. Figure S5: Time-resolved UV-Vis spectra of phenol during Vis irradiation in the presence of (a) Co(0.1%)/TiO_2_, (b) Co(0.3%)/TiO_2_, (c) Co(0.5%)/TiO_2_, (d) Co(1%)/TiO_2_. [Phenol] = 50 ppm; Photocatalyst = 1g·L^−1^, T ca. 25 °C, natural pH. Table S1: Raman peak centred at 143 cm^−1^ as a function of laser line and laser’s power. Table S2: Band gap for TiO_2_-P25 and Co doped TiO_2_ photocatalysts synthesized by different methods.
