# Supplementary material for: Cobalt Impregnation on Titania Photocatalysts Enhances Vis Phenol Photodegradation
Source: Materials (Basel). 2023 Jun 1;16(11):4134. doi: 10.3390/ma16114134 (PMC10254245; doi:10.3390/ma16114134)
Supplement: Supplementary file 1 [file materials-16-04134-s001.zip › materials-2325613-supplementary.pdf]

Supplementary Information

Article

Cobalt Impregnation on Titania Photocatalysts Enhances Vis Phenol Photodegradation

Soukayna Belekbir [1], Mohammed El Azzouzi [1], Laura Rodríguez-Lorenzo [2], Adnane El Hamidi [1], Juan Arturo Santaballa [3,*] and Moisés Canle [3,*]

[1] Laboratory of Nanomaterials, Nanotechnologies and Environment, Center of Materials Science, Faculty of Sciences, Mohammed V University in Rabat, Rabat BP 1014, Morocco
[2] INL—International Iberian Nanotechnology Laboratory, Water Quality Group, Av. Mestre José Veiga, 4715-330 Braga, Portugal
[3] React! Group, Department of Chemistry, Faculty of Sciences & CICA, University of A Coruña, E-15071 A Coruña, Spain
* Correspondence: arturo.santaballa@udc.es (J.A.S.); moises.canle@udc.es (M.C.)

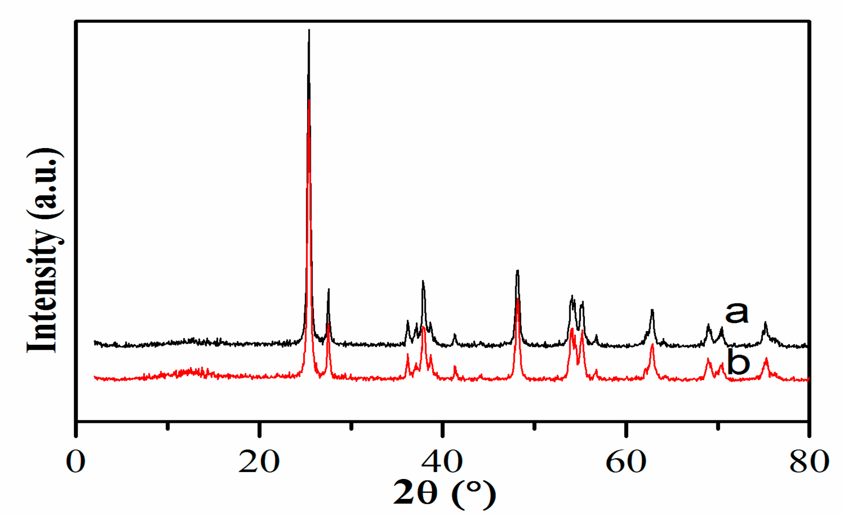

Figure S1. Diffractograms of Co(0.1%)/TiO_2, a: before leaching test, b**:** after leaching test.

Table S1: Raman peak centred at 143 cm^{-1} as a function of laser line and laser's power.

Laser power	***Laser line***	**633 nm**		**785 nm**	
	Photocatalyst	**P25**	**Co(0.1%)/TiO_2**	**P25**	**Co(0.1%)/TiO_2**
**21-28 mW**	*Peak position (cm^{-1})*	141	141	144	144
	Height (CCD cts)	10160	12954	9620	18381
	FWHM (cm^{-1})	4.34	19.7	14.1	14.4
**10 mW**	*Peak position (cm^{-1})*	141	141	n.a.	n.a.
	Height (CCD cts)	1304	2250	n.a.	n.a.
	FWHM (cm^{-1})	15.07	15.63	n.a.	n.a.
**35 mW**	*Peak position (cm^{-1})*	n.a.	n.a.	144	144
	Height (CCD cts)	n.a.	n.a.	15831	45907
	FWHM (cm^{-1})	n.a.	n.a.	13.9	14.0

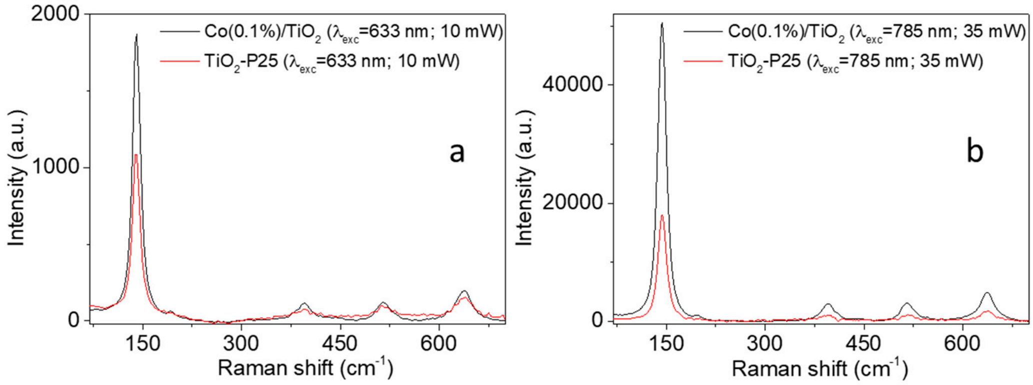

Figure S2: Raman spectra of TiO_2-P25 and Co(0.1%)/TiO_2 acquired using (a) 633 nm and (b) 785 nm excitation laser line.

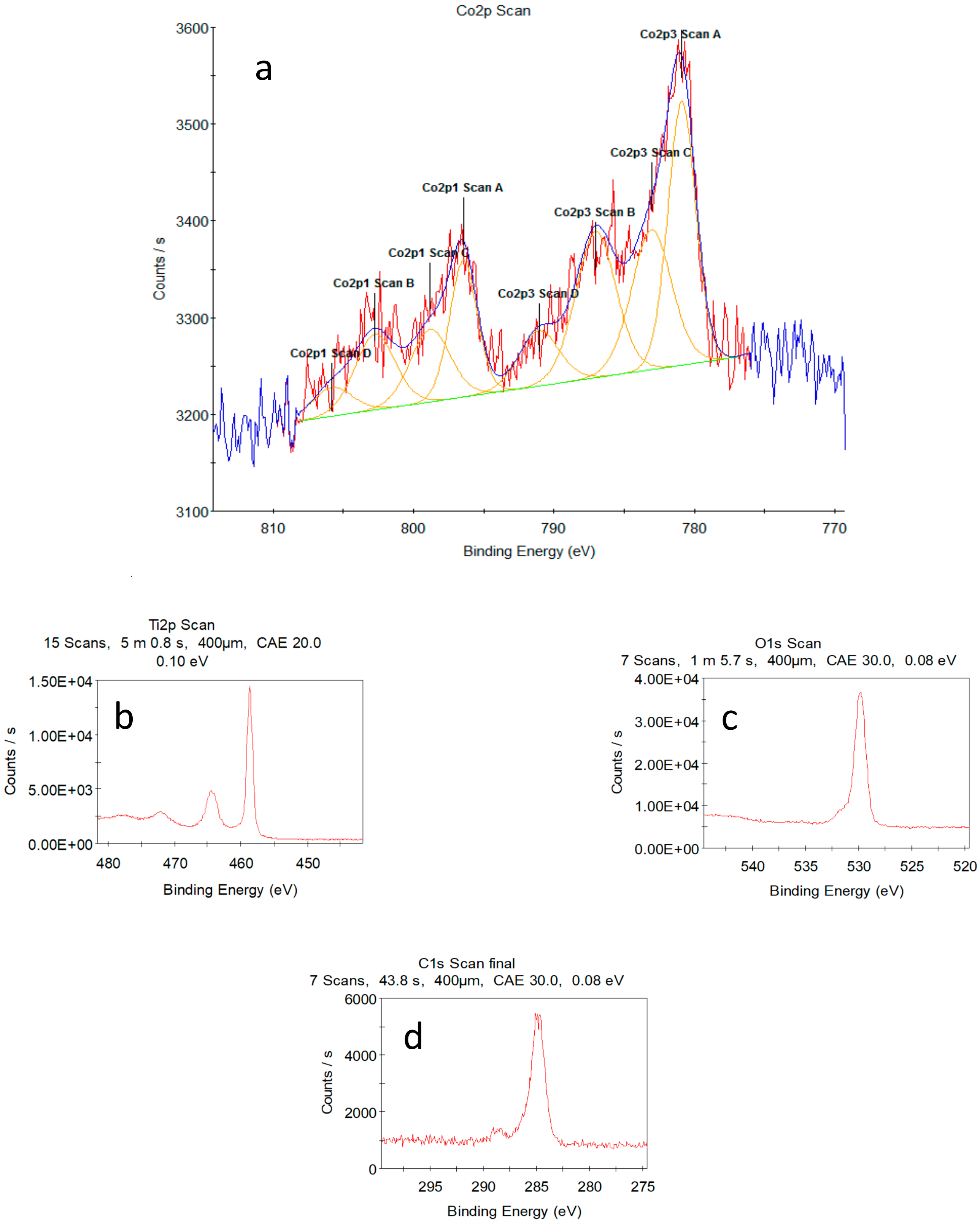

Figure S3. XPS spectra (a) Co 2p scan and fitting; (b) Ti 2p scan; (c) O 1s scan; (d) C 1s scan.

Table S2: Band gap for TiO_2-P25 and Co doped TiO_2 photocatalysts synthesized by different methods.

Photocatalyst	Co (%wt)	Synthetic method	Band gap (eV)	Reference (DOI)
TiO_2-P25	--	--	3.30	This work
Co/TiO_2	0.1	Impregnation	2.4	This work
Co/TiO_2	0.1	Sol-gel	2.66	10.1007/s10971-015-3715-3
Co/TiO_2	0.1	Sol-gel	2.77	10.3390/ma11101946
Co/TiO_2	0.1	Coprecipitation	2.78	10.1016/j.matchemphys.2012.04.062
Co/TiO_2	0.3	Impregnation	2.3	This work
Co/TiO_2	0.3	Sol-gel	3.00	10.5004/dwt.2017.20205
Co/TiO_2	0.5	Impregnation	2.3	This work
Co/TiO_2	0.5	Sol-gel	2.48	10.1016/j.sajce.2017.10.001
Co/TiO_2	0.5	Hydrothermal	3.19	10.1016/j.apcatb.2016.03.003<br>1-s2.0-S0926337316301692-main
Co/TiO_2	0.5	Sol-gel & Precipitation	3.24	10.1016/j.chemosphere.2020.128931
Co/TiO_2	0.67	Sol-gel	3.02	10.1016/j.jwpe.2017.02.015<br>1-s2.0-S2214714416305256-main
Co/TiO_2	1.0	Impregnation	2.3	This work
Co/TiO_2	1.0	Sol-gel	1.97	10.1007/s10971-015-3715-3
Co/TiO_2	1.0	Hydrothermal	3.13	0.1016/j.apcatb.2016.03.003<br>1-s2.0-S0926337316301692-main
Co/TiO_2	1.0	Sol-gel & Precipitation	3.05	10.1016/j.chemosphere.2020.128931

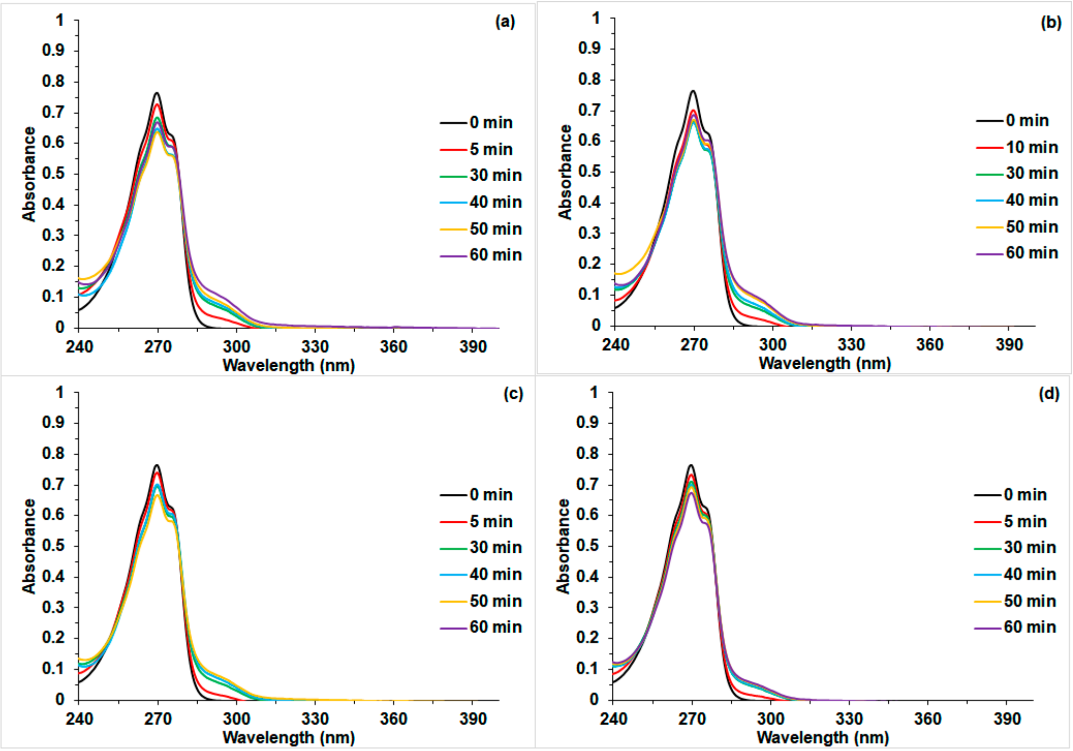

Figure S4: Time-resolved UV-Vis spectra of phenol during its UV irradiation in the presence of (a) Co(0.1%)/TiO_2, (b) Co(0.3%)/TiO_2, (c) Co(0.5%)/TiO_2, (d) Co(1%)/TiO_2. [Phenol] = 50 ppm; Photocatalysts = 1g·L^{-1}, T *ca.* 25^0C, natural pH.

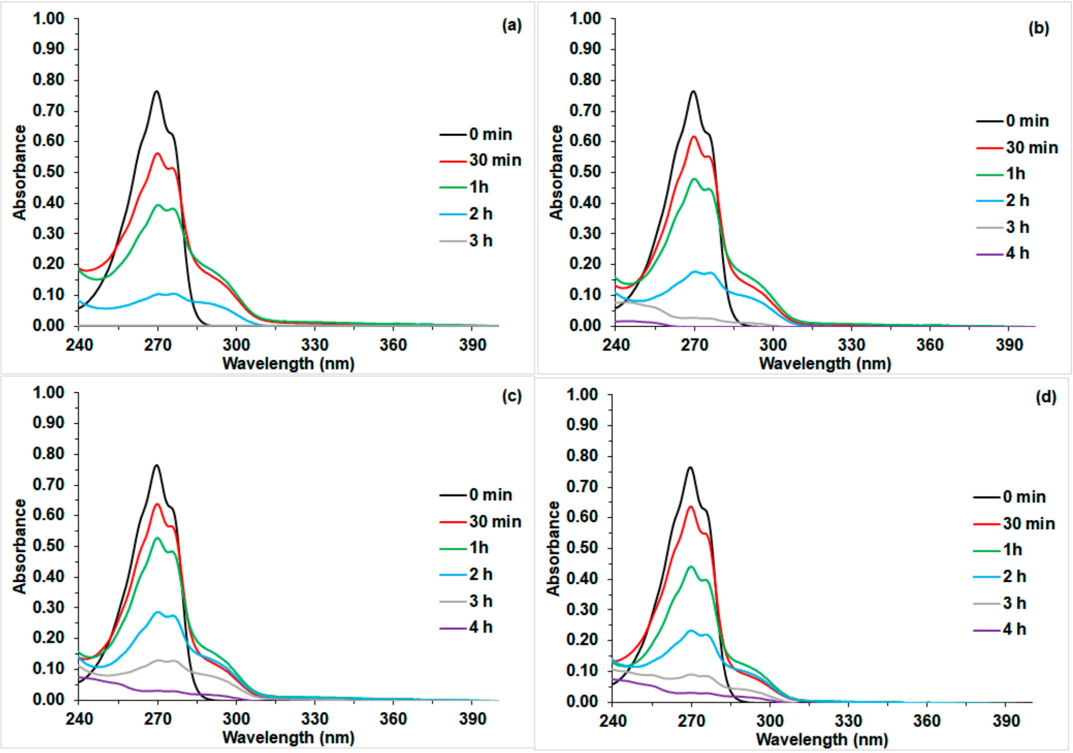

Figure S5: Time-resolved UV-Vis spectra of phenol during Vis irradiation in the presence of (a) Co(0.1%)/TiO_2, (b) Co(0.3%)/TiO_2, (c) Co(0.5%)/TiO_2, (d) Co(1%)/TiO_2. [Phenol] = 50 ppm; Photocatalyst = 1g·L^{-1}, T *ca.* 25^0C, natural pH.
